# Microsurgical clipping of an unruptured superiorly projecting ACoM aneurysm in a rotated ACoM complex

**DOI:** 10.1002/ccr3.2022

**Published:** 2019-03-19

**Authors:** Gregory Glauser, Omar A. Choudhri

**Affiliations:** ^1^ Department of Neurosurgery Hospital of the University of Pennsylvania Philadelphia Pennsylvania

**Keywords:** ACoM aneurysm, aneurysm clipping, cerebrovascular, unruptured aneurysm repair

## Abstract

This case illustrates surgical technique for wide neck aneurysm clipping in a rotated complex and how to manage intraoperative aneurysm rupture while maintaining hemostasis (Cai et al., Anterior communicating artery aneurysm morphology and the risk of rupture. World Neurosurg. 2018;109:119 and Dehdashti et al., The implication of anterior communicating complex rotation and 3‐dimensional computerized tomography angiography findings in surgical approach to anterior communicating artery aneurysms. World Neurosurg. 2016;91:34).

This case is a presentation of one possible approach for repair of an unruptured rotated anterior communicating artery aneurysm.^1^, ^2^ The patient presented with headaches and was found to have a 6 mm anterior communicating artery aneurysm. The case shows how to manage intraoperative aneurysm rupture while maintaining hemostasis.

## ETHICS STATEMENT

This video was deemed IRB exempt by the Institutional Review Board (IRB) as it is considered a case report, which does not require IRB approval or patient consent. All ethical rules and guidelines were followed.

## CONFLICT OF INTEREST

None declared.

## AUTHOR CONTRIBUTION

OC: performed the procedure and provided video narration. GG: performed critical video editing and preparation for publication.
